# DIAGnosis using NOvel technology for Subtypes In Stroke (DIAGNOSIS study): performance evaluation protocol

**DOI:** 10.1136/bmjno-2026-001575

**Published:** 2026-09-03

**Authors:** Ibrahim Alghamdi, Mohammed Almubayyidh, Edoardo Gaude, David A Jenkins, Adrian R Parry-Jones

**Affiliations:** 1 Division of Cardiovascular Sciences, School of Medical Sciences, Faculty of Biology, Medicine and Health, The University of Manchester, Manchester, UK; 2 Department of Emergency Medical Services, College of Applied Medical Sciences, King Khalid University, Abha, Saudi Arabia; 3 Prince Sultan Bin Abdulaziz College for Emergency Medical Services, King Saud University, Riyadh, Saudi Arabia; 4 Upfront Diagnostics, Pockit Diagnostics Ltd, Cambridge, UK; 5 Division of Informatics Imaging and Data Science, The University of Manchester, Manchester, UK; 6 Geoffrey Jefferson Brain Research Centre, Manchester Academic Health Science Centre, Northern Care Alliance, Salford, UK

**Keywords:** NEUROPATHOLOGY, NEUROPATHY, NEUROUROLOGY, STROKE

## Abstract

**Introduction:**

Timely and accurate identification of stroke subtypes, particularly intracerebral haemorrhage (ICH) and large vessel occlusion (LVO), is essential for improving stroke outcomes. Biomarkers have shown potential in identifying these subtypes. Glial fibrillary acidic protein (GFAP), which is elevated in ICH, and D-dimer, which is associated with LVO, are promising for rapid, point-of-care identification. This study evaluates whether combining GFAP and D-dimer with clinical features improves the diagnostic accuracy for ICH and LVO.

**Methods and analysis:**

This is a multicentre, observational diagnostic accuracy study recruiting a minimum of 257 patients with suspected stroke within 6 hours of symptom onset from two UK hyperacute stroke centres. The study will integrate clinical features and point-of-care biomarker results using the LVOne test (GFAP and D-dimer) to develop diagnostic models that differentiate ICH and LVO from other stroke presentations. Patients will be identified on hospital arrival, and clinical variables and blood samples collected immediately, with the final diagnosis recorded prior to discharge. Statistical analyses will assess discrimination, calibration and clinical utility, with sensitivity, specificity and predictive values reported. Model development and reporting will follow the Transparent Reporting of a Multivariable Prediction Model for Individual Prognosis or Diagnosis+Artificial Intelligence and the Standards for Reporting of Diagnostic Accuracy Studies guidelines.

**Ethics and dissemination:**

The study is registered with ISRCTN (ISRCTN35174477). Ethical approval has been obtained from the Yorkshire & The Humber – Leeds West Research Ethics Committee (25/YH/0102) and the Health Research Authority. Results will be disseminated via open-access peer-reviewed journals, presentations at national and international conferences and summaries for patient and public contributors.

**WHAT IS ALREADY KNOWN ON THIS TOPIC**

- Existing prehospital tools, such as FAST (Face, Arm, Speech Test), are designed for undifferentiated stroke recognition and are not intended to distinguish intracerebral haemorrhage (ICH) or large vessel occlusion (LVO).
- Brain-specific biomarkers, such as glial fibrillary acidic protein (GFAP) and D-dimer, have shown promise in differentiating stroke subtypes, but most studies have assessed them in isolation or outside of real-world emergency care pathways.

**WHAT THIS STUDY ADDS**

- This multicentre study will evaluate the diagnostic performance of the LVOne test, a point-of-care lateral flow platform measuring GFAP and D-dimer, in combination with routinely available clinical features, in patients with suspected stroke within 6 hours of onset.
- The study will determine whether integrating GFAP and D-dimer with clinical features into diagnostic models improves diagnostic accuracy for differentiating ICH and LVO.

**HOW THIS STUDY MIGHT AFFECT RESEARCH, PRACTICE OR POLICY**

- If the models show adequate performance, they will inform the design of future prehospital validation studies and could ultimately support earlier, more accurate triage of suspected stroke patients.
- The findings of this study may help refine ambulance destination decisions and prehospital treatment strategies, such as blood pressure lowering in ICH or direct transfer of patients with LVO to thrombectomy-capable centres.

## Introduction

 In the UK, ambulance services commonly use the Face, Arm, Speech Test (FAST) for stroke recognition.^1–3^ However, tools such as FAST were developed to detect undifferentiated stroke and were not intended to identify intracerebral haemorrhage (ICH) and large vessel occlusion (LVO).^4–6^

Among stroke subtypes, ICH and LVO are particularly severe. Timely treatment, such as blood pressure lowering to limit haematoma expansion in ICH or thrombectomy for LVO, is critical^7 8^ and has shifted the focus to the prehospital phase. The Intensive Ambulance-Delivered Blood Pressure Reduction in Hyper-Acute Stroke Trial (INTERACT4) showed that intensive blood pressure lowering initiated in the ambulance significantly reduced death and disability at 90 days in patients later diagnosed with ICH, but worsened outcome in those with ischaemic stroke (IS), including LVO.^9^ The Effect of Direct Transportation to Thrombectomy-Capable Centre vs Local Stroke Centre on Neurological Outcomes in Patients With Suspected Large-Vessel Occlusion Stroke in Nonurban Areas Trial (RACECAT) found that bypassing the nearest stroke centre to transport suspected stroke patients directly to a thrombectomy-capable centre was associated with worse outcomes in patients ultimately diagnosed with ICH.^10^ These findings underscore the importance of distinguishing stroke subtypes in the ambulance to guide appropriate prehospital care and destination decisions.

To realise this ambition, a reliable method that can quickly and accurately differentiate between stroke subtypes in the prehospital setting is required. Although there has been considerable interest in prehospital scales based on clinical features for this purpose,^11–13^ concerns remain regarding their real-world accuracy when used alone.^14^ Recently, innovative technologies, such as brain-specific biomarker testing, have emerged as potentially cost-effective and non-invasive approaches to facilitate early diagnosis and treatment in patients with suspected stroke.^15^ Among these biomarkers, glial fibrillary acidic protein (GFAP), an astrocyte marker that rises early after ICH, and D-dimer, a fibrin degradation product indicative of intravascular coagulation, have shown promise in distinguishing between stroke subtypes.^16 17^

A pooled analysis of 12 studies demonstrated that GFAP could differentiate ICH from IS and stroke mimics, with a sensitivity of 78% (95% CI 67 to 86%) and a specificity of 95% (95% CI 88 to 98%).^16^ Furthermore, a study by Ramos-Pachón *et al*^17^ found that D-dimer was associated with LVO and, when combined with a National Institutes of Health Stroke Scale (NIHSS) score of ≥10, detected LVOs with 35% sensitivity and 93% specificity. These findings suggest that combining GFAP and D-dimer with clinical features could help identify ICH and LVO with sufficient diagnostic accuracy to assist prehospital clinicians in making crucial decisions regarding therapeutic interventions (eg, lowering blood pressure for patients with ICH) and determining appropriate transport destinations.

The aim of this study is to investigate whether diagnostic models based on clinical features combined with point-of-care GFAP and D-dimer tests can accurately determine stroke subtypes in patients with suspected stroke presenting to participating hospitals within 6 hours of symptom onset.

## Study objectives

### Primary objective

To develop and validate diagnostic models that combine clinical features with results from UpFront Diagnostics’ GFAP and D-dimer point-of-care lateral flow tests (collectively referred to as the ‘LVOne test’) to identify ICH and LVO in adults with suspected stroke presenting within 6 hours of symptom onset.

### Secondary objectives

- To estimate the diagnostic accuracy of GFAP and D-dimer, individually and in combination, for identifying ICH and LVO.
- To compare the performance of models based on clinical features alone with models that additionally incorporate LVOne biomarker results.
- To describe the feasibility and timing of point-of-care biomarker sampling in the early in-hospital phase.

If successful, and if the models demonstrate adequate performance, this work will provide a foundation for future prospective validation studies in the prehospital setting and could ultimately support a shift from generic prehospital stroke recognition towards early differentiation of stroke subtypes, enabling more targeted treatment and triage (eg, direct transport of patients with LVO to thrombectomy-capable centres and prehospital blood pressure lowering in ICH).

## Study design

DIAGnosis using NOvel technology for Subtypes In Stroke (DIAGNOSIS) is a two-centre prospective diagnostic accuracy study of patients with suspected stroke presenting within 6 hours of symptom onset to two hyperacute stroke centres in Greater Manchester (GM). The diagnostic accuracy of the models will be evaluated against final diagnoses established by CT and/or MRI of the brain.

### Study procedures

Recruitment will take place at two major stroke centres in GM: Salford Royal Hospital and Fairfield General Hospital. At each participating site, a member of the direct clinical care team will approach suspected stroke patients within 6 hours of symptom onset on arrival to conduct initial screening and identification.

If the patient is deemed to meet the eligibility criteria on arrival, a finger-prick capillary blood sample will be immediately collected (within 6 hours of symptom onset) from the patient for the LVOne test. The sample will then be provided to the research team for testing, with the clinical care team remaining blinded to the result to avoid any possible changes to clinical care. A venous blood sample will be collected at the same time for laboratory measurement of GFAP and D-dimer. The sample will be processed as soon as possible but no later than 1 hour after collection.

Once emergency assessments and treatments have been completed, and after all LVOne testing and baseline blood sampling have been performed within the 6 hour onset window, patients will be approached for enrolment and written consent no later than 72 hours after hospital arrival. If the patient has capacity, they will be provided with a participant information sheet and a verbal explanation, and given adequate time to ask questions before written consent is obtained.

Since patients with suspected stroke frequently lack capacity, and assessments must be conducted urgently, it is not always feasible to seek prospective informed consent before LVOne testing and blood sampling. A deferred consent approach will therefore be used in accordance with the UK Mental Capacity Act 2005. Patients who regain capacity within 72 hours of hospital arrival will be approached for written informed consent. For those who remain unable to consent, the opinion of a personal consultee (such as a relative or close friend) will be sought, or, if none is available, the opinion of a nominated (professional) consultee independent of the study team. If neither consent nor the opinion of a consultee is obtained within 72 hours, all study samples and LVOne results for that individual will be destroyed and no further data will be collected.

Routinely collected prehospital and hospital clinical data, as well as discharge diagnosis and treatment information will be extracted from the medical records.

### Justification for emergency consent process

To simulate prehospital conditions and support early diagnostic model development, samples will be collected immediately on hospital arrival, including finger-prick and venous blood samples under an ethically approved deferred consent process. Early sampling is critical, as both clinical features and biomarker levels change rapidly following stroke onset; timely collection ensures scientific validity by capturing the early pathophysiological state. While early clinical data can be retrospectively extracted from medical records after consent, biomarker testing must be performed promptly at presentation.

Finger-prick sampling for the LVOne test is minimally invasive, consistent with routine clinical practice (eg, capillary blood glucose testing), and will be performed by clinical staff who remain blinded to the result. Venous blood samples will be taken alongside routine clinical draws where feasible to avoid additional venepuncture and minimise patient burden. If this is not possible, a separate research sample will be collected as soon as possible by the clinical team following verbal consent. This approach enables timely data collection without delaying urgent clinical care, while maintaining ethical safeguards and patient comfort.

### Justification for hospital-based design

The study was designed as a hospital-based evaluation as an initial step in the clinical validation of the LVOne device. Before implementation in the prehospital setting, it is important to establish the feasibility, diagnostic performance and operational characteristics of the test within a controlled clinical environment, where standard reference diagnostics and clinical oversight are readily available.

Conducting the study in the hospital setting allows for timely blood sampling in the hyperacute phase, close alignment with routine clinical workflows and direct comparison with imaging-based diagnoses. It also enables concurrent collection of venous blood samples for laboratory-based assays, minimising additional procedures and patient burden while facilitating assessment of analytical agreement.

Implementation in the prehospital setting would require additional considerations and substantial resources, including training a large number of ambulance personnel, integration into time-critical emergency workflows and system-level coordination across ambulance services. In addition, individual ambulance clinicians may encounter relatively few eligible stroke cases, meaning that a large number of prehospital providers and sites would be needed to achieve the required sample size within a reasonable timeframe. For these reasons, we chose to evaluate LVOne in a hospital-based setting first. These factors would substantially increase study complexity at this stage and are more appropriately addressed following initial clinical validation.

To approximate the intended use in the prehospital pathway and to reflect the urgency and time-sensitive nature of the prehospital phase, blood sampling and testing will be performed as early as possible following hospital arrival and within 6 hours of symptom onset. If the device and resulting models demonstrate adequate diagnostic performance in this setting, the next stage will be to evaluate LVOne in the prehospital environment, and we will seek funding to support implementation and testing in ambulances and other prehospital services.

### Description of the LVOne test and biomarker assays

The LVOne is a single-use point-of-care test comprising two portable lateral flow assays: one for detecting D-dimer and the other for detecting GFAP. Each assay provides a semi-quantitative estimate of biomarker concentration based on test-line intensity. In each assay, a blood sample is mixed with an antibody-nanoparticle conjugate and applied to the sample port. Following the addition of a reaction buffer, the resulting complex moves along the membrane and binds to a capture antibody, producing a coloured test line whose visual intensity increases with biomarker concentration.

Test validity is determined by the presence of both GFAP and D-dimer control lines. Tests in which either control line is absent are classified as invalid. Test-line intensity for GFAP and D-dimer is scored on an ordinal 0–10 scale (0=no visible test line; 1–10=increasing band intensity), using the manufacturer’s reference card. The concentration ranges represented by the reference card are 213–30 000 pg/mL for GFAP and 400–15 000 ng/mL for D-dimer. A GFAP test-line intensity score of ≥1 corresponds to a concentration of ≥213 pg/mL, while a D-dimer test-line intensity score of ≥2 corresponds to a concentration of ≥600 ng/mL. In accordance with manufacturer-prespecified thresholds, GFAP is considered positive if any visible test line is present (intensity score ≥1) and negative if the intensity score is 0. D-dimer is considered positive if the test-line intensity score is ≥2, and negative if the intensity score is 0 or 1.

All LVOne tests will be interpreted at the point of care by a trained primary reader (Reader 1), who will record control line validity and intensity scores for each biomarker using a standardised procedure and reference card. Where available, a second independent reader, blinded to the primary reader, will also record control-line validity and intensity scores to allow assessment of inter-reader agreement.

Stroke subtype classification using the LVOne will be performed in accordance with the manufacturer’s LVOne intended use and the prespecified combined clinical-biomarker rule to classify patients into haemorrhagic stroke or LVO. Haemorrhagic stroke will be defined as FAST ≥1 and GFAP positive (intensity ≥1), irrespective of D-dimer. LVO will be defined as FAST ≥2, GFAP negative (intensity=0) and D-dimer positive (intensity ≥2). All other combinations will be classified as non-haemorrhagic and non-LVO. These LVOne stroke subtype classifications will not be performed by clinical staff in real time; instead, they will be derived analytically by the research team in the analysis phase at the end of the study based on the recorded FAST scores and LVOne intensity readings.

An enzyme-linked immunosorbent assay (ELISA) will also be used to quantify GFAP and D-dimer concentrations. Results from the ELISA will be compared with those from the lateral flow assays to assess analytical agreement. If GFAP and D-dimer alone are insufficient to distinguish stroke subtypes with adequate accuracy, additional plasma biomarkers previously shown to differentiate between stroke, stroke mimics, and specific subtypes may also be explored.

### Venous blood sample processing, analysis and storage

A 4-mL venous blood sample will be drawn into an acid citrate dextrose anticoagulant tube as soon as possible after hospital arrival and always within 6 hours of symptom onset. Samples will be stored at 4 °C and transported to the research laboratory, where plasma will be separated by centrifugation within 1 hour of collection, aliquoted and stored at −80 °C.

Plasma aliquots will be stored on site until transfer to the central laboratory (UpFront Diagnostics) for analysis of GFAP and D-dimer. Following study completion, any remaining samples will be stored for future ethically approved research, subject to specific additional consent from participants.

### End of study

The end of the study will be defined by the study end date, or the recruitment of at least 257 participants, including a minimum of 26 patients with ICH and 26 with LVO, whichever occurs first.

## Study participants

### Inclusion criteria

- The patient arrived at the study hospital via emergency ambulance.
- The patient is 18 years of age or older.
- The ambulance staff suspected a new acute stroke prior to arrival at the hospital.
- Stroke symptoms began within 6 hours of research sample collection (if unwitnessed onset, patient was last known to be well within 6 hours).
- Blood samples can be collected prior to the administration of thrombolysis or any other acute treatment.
- Urgent brain imaging is planned in line with national stroke guidelines.

### Exclusion criteria

- The patient was assessed at another hospital and transferred for ongoing care.
- The patient had a recent diagnosis (within the preceding 4 weeks) of deep vein thrombosis, pulmonary embolism, arterial embolism, stroke, transient ischaemic attack, long bone fracture, major trauma or surgery under general anaesthesia (which may increase D-dimer levels).
- The patient had a recent head injury (within the preceding 4 weeks) requiring hospital care (which may increase GFAP levels).
- Stroke symptoms started more than 6 hours prior to sample collection.

### Patients who withdraw consent

If a patient chooses to withdraw from the study, any data collected (LVOne test results, blood samples and clinical data) will be securely destroyed. However, as data will be anonymised as soon as possible following study completion, it will not be possible to remove information once anonymisation has occurred, since it will no longer be traceable to individual participants.

### Patient and public involvement

The DIAGNOSIS study was reviewed by the Geoffrey Jefferson Brain Research Centre Patient, Carer and Public Involvement group. Members supported the study’s aims and endorsed the deferred consent model given the time-critical nature of acute stroke care. Their feedback informed the approach to early sampling and consent process.

## Reference standard

The final in-hospital diagnosis of stroke subtype, as determined by stroke consultants or senior physicians responsible for the patient’s care, will serve as the reference standard. Diagnoses will be based on clinical assessment, brain imaging (CT and/or MRI), laboratory findings and other relevant examinations. Treating clinicians will be blinded to the LVOne test and research biomarker results. Where diagnostic uncertainty arises, the case will be reviewed and adjudicated by the site principal investigator, who is a senior stroke consultant. If the site principal investigator’s opinion differs from the clinical team’s diagnosis, a third independent senior stroke clinician will be asked to adjudicate.

For each participant, the final diagnosis will be recorded as IS, ICH or a stroke mimic, and in cases of IS, the presence of LVO will be recorded.

## Data collection, source data and confidentiality

For patients who provide consent, study data will be recorded on a case report form and entered into a secure study database hosted at the University of Manchester. Collected variables will include demographics (eg, age and sex), presenting symptoms (eg, unilateral weakness and speech disturbance), prehospital and admission vital signs (eg, blood pressure, heart rate and oxygen saturation), past medical history, and details of blood sampling and LVOne test line status.

From the ambulance patient report form, prehospital stroke scale assessments (eg, FAST) and their components (face weakness, arm weakness and speech disturbance) will be extracted, together with other presenting symptoms, including those typical of posterior circulation stroke (eg, vertigo, ataxia and visual disturbance).

Key time points, including symptom onset or last known well, first paramedic contact, hospital arrival, and LVOne and venous sampling will be recorded to calculate onset-to-sample and prehospital-to-sample intervals.

Data will be collected from the ambulance patient report form, as well as from assessments and records in the emergency department. During follow-up, additional information will be obtained, including final diagnosis, brain imaging findings and details of acute treatments.

All brain imaging performed during the acute admission will be pseudonymised and transferred to the DIAGNOSIS research team at the University of Manchester for further analysis. These scans will be used to confirm stroke subtype and to characterise lesion location, size and other imaging features relevant to clinical presentation and biomarker levels.

## Statistical considerations

### Statistical analysis

The statistical analysis plan will be finalised prior to analysis in collaboration with the lead study statistician. Multivariable regression will be used to develop diagnostic models based on the collected data, and other statistical or machine learning methods will be considered where appropriate.

The set of clinical predictors to be included in the models will be informed by prior literature and clinical plausibility and will not be selected solely on the basis of univariable associations. The number of candidate predictors will be guided by sample size criteria proposed by Riley *et al*^18 19^ to minimise overfitting and ensure reliable estimation in diagnostic prediction models.

Model development will follow best practices described by Steyerberg,^20^ incorporating internal validation via bootstrapping to estimate and adjust for optimism in model performance.

Model performance will be assessed in terms of discrimination (area under the receiver operating characteristic curve), calibration (using calibration plots and calibration slope and intercept) and clinical utility using decision curve analysis.^21^ Sensitivity, specificity and predictive values will also be reported with 95% CIs.

Model development and reporting will adhere to the Transparent Reporting of a Multivariable Prediction Model for Individual Prognosis or Diagnosis+Artificial Intelligence guidelines.^22^ Analyses of biomarkers performed separately will be reported in accordance with the Standards for Reporting of Diagnostic Accuracy Studies guidelines to ensure a transparent and complete evaluation of diagnostic accuracy.^23^ While we will lack the power to test for heterogeneity by subgroup, exploratory secondary analysis will be considered, including testing by onset-to-test time of 0–2 hours vs 2–6 hours.

### Missing data

Missing data in clinical variables and laboratory biomarker measurements may arise due to incomplete documentation or unsuccessful sample collection. Such data will be assessed and handled using appropriate statistical methods in line with best practice for prediction model development. Where appropriate, multiple imputation will be used under the assumption that data are missing at random. Participants with missing LVOne results will be excluded from the primary analysis. Sensitivity analyses will be conducted, where appropriate, to evaluate the potential impact of missing data on model development and performance.

### Sample size

This study aims to ‘rule in’ patients with ICH and LVO; therefore, the sample size calculation prioritises high specificity.

Assuming a 10% prevalence of both ICH and LVO, a sample size of 257 patients with suspected stroke is required, including a minimum of 26 confirmed cases for each subtype. The sample size calculation is based on a diagnostic accuracy framework using the method described by Bujang and Adnan,^24^ in which null and alternative hypotheses are specified for sensitivity and specificity.

Based on our previous work, which demonstrated a specificity of 90% and a sensitivity of 50% for a clinical model with nine predictors,^25^ the current study is powered to detect an improvement to 95% specificity and 80% sensitivity for the detection of ICH and LVO, with 80% power and a significance level of 0.05.^24^

The number of cases contributing to the primary analysis will be monitored throughout the study. If necessary, recruitment may be adjusted to ensure adequate representation of target subtypes.

## Monitoring and quality assurance

The study will be subject to internal monitoring led by the Chief Investigator to ensure it is conducted in accordance with the approved protocol, good clinical practice (GCP) and applicable regulatory standards. Routine monitoring activities may include a review of informed consent documentation, verification of source data and assessment of compliance with study procedures at participating sites.

## Safety considerations and adverse events

In this observational study, risks to participants are expected to be minimal. Research blood samples will be collected alongside routine clinical samples where possible. When this is not feasible, additional venous samples may be obtained following verbal consent. Finger-prick sampling for the LVOne test may cause minor discomfort or bruising; however, these effects are rare and transient. All procedures will be performed by trained clinical staff.

All events, including adverse events and serious adverse events, considered by the local team to be potentially related to the device will be recorded. Serious adverse device effects will be reported by the Chief Investigator or Principal Investigator within 24 hours to the study sponsor (The University of Manchester) and the host hospital research and development department.

## Peer review

Funding for the study was awarded through the Confidence for Translation (C4T) scheme at the University of Manchester, and the funding application was reviewed by six independent scientific experts at the University of Manchester. In addition, the protocol was reviewed by two expert stroke academics from other institutions, independent of the DIAGNOSIS study team.

## Ethics and dissemination

### Sponsorship and ethical approvals

The study is sponsored by the University of Manchester and registered with the ISRCTN registry (ISRCTN35174477). Ethical approval has been obtained from the Yorkshire & The Humber - Leeds West Research Ethics Committee (reference 25/YH/0102), the Health Research Authority, and the participating NHS institutions.

The research will be conducted in accordance with the principles of GCP and the Declaration of Helsinki. LVOne is an in vitro diagnostic medical device that is UKCA-marked for the identification of LVO. In this study, it will also be used to evaluate its diagnostic accuracy and potential clinical utility in identifying ICH and stroke mimics, indications for which it is not currently UKCA-marked. Consequently, the study is classified as a ‘performance evaluation’, and the Medicines and Healthcare products Regulatory Agency (MHRA) has been notified of the study prior to the commencement of recruitment.

Study findings will be disseminated through open-access peer-reviewed journals, presentations at national and international conferences and feedback to participating sites and to patient and public contributors. De-identified data may be shared with other researchers on reasonable request, subject to sponsor and data governance approvals.

## Data Availability

Data sharing not applicable as no datasets generated and/or analysed for this study.

## References

[1] Fothergill RT, Williams J, Edwards MJ (2013). Does Use of the Recognition Of Stroke In the Emergency Room Stroke Assessment Tool Enhance Stroke Recognition by Ambulance Clinicians?. Stroke.

[2] Kleindorfer DO, Miller R, Moomaw CJ (2007). Designing a message for public education regarding stroke: does FAST capture enough stroke?. Stroke.

[3] Harbison J, Hossain O, Jenkinson D (2003). Diagnostic accuracy of stroke referrals from primary care, emergency room physicians, and ambulance staff using the face arm speech test. Stroke.

[4] Oostema JA, Chassee T, Baer W (2021). Accuracy and Implications of Hemorrhagic Stroke Recognition by Emergency Medical Services. Prehosp Emerg Care.

[5] Shaw L, Graziadio S, Lendrem C (2021). Purines for Rapid Identification of Stroke Mimics (PRISM): study protocol for a diagnostic accuracy study. Diagn Progn Res.

[6] Gibson LM, Whiteley W (2013). The differential diagnosis of suspected stroke: a systematic review. J R Coll Physicians Edinb.

[7] Bowry R, Parker SA, Bratina P (2022). Hemorrhage Enlargement Is More Frequent in the First 2 Hours: A Prehospital Mobile Stroke Unit Study. Stroke.

[8] Saver JL, Goyal M, van der Lugt A (2016). Time to Treatment With Endovascular Thrombectomy and Outcomes From Ischemic Stroke: A Meta-analysis. JAMA.

[9] Li G, Lin Y, Yang J (2024). Intensive Ambulance-Delivered Blood-Pressure Reduction in Hyperacute Stroke. N Engl J Med.

[10] Ramos-Pachón A, Rodríguez-Luna D, Martí-Fàbregas J (2023). Effect of Bypassing the Closest Stroke Center in Patients with Intracerebral Hemorrhage: A Secondary Analysis of the RACECAT Randomized Clinical Trial. JAMA Neurol.

[11] Geisler F, Wesirow M, Ebinger M (2021). Probability assessment of intracerebral hemorrhage in prehospital emergency patients. Neurol Res Pract.

[12] Uchida K, Yoshimura S, Hiyama N (2018). Clinical Prediction Rules to Classify Types of Stroke at Prehospital Stage. Stroke.

[13] Jin H-Q, Wang J-C, Sun Y-A (2016). Prehospital Identification of Stroke Subtypes in Chinese Rural Areas. Chin Med J (Engl).

[14] Greenberg SM, Ziai WC, Cordonnier C (2022). 2022 Guideline for the Management of Patients With Spontaneous Intracerebral Hemorrhage: A Guideline From the American Heart Association/American Stroke Association. Stroke.

[15] Kaffes M, Bondi F, Geisler F (2023). Optimization of sensitivity and specificity of a biomarker-based blood test (LVOCheck-Opti): A protocol for a multicenter prospective observational study of patients suspected of having a stroke. Front Neurol.

[16] Kumar A, Misra S, Yadav AK (2020). Role of glial fibrillary acidic protein as a biomarker in differentiating intracerebral haemorrhage from ischaemic stroke and stroke mimics: a meta-analysis. Biomarkers.

[17] Ramos-Pachón A, López-Cancio E, Bustamante A (2021). D-Dimer as Predictor of Large Vessel Occlusion in Acute Ischemic Stroke. Stroke.

[18] Riley RD, Snell KI, Ensor J (2019). Minimum sample size for developing a multivariable prediction model: PART II - binary and time-to-event outcomes. Stat Med.

[19] Riley RD, Ensor J, Snell KIE (2020). Calculating the sample size required for developing a clinical prediction model. BMJ.

[20] Steyerberg EW (2019). Clinical Prediction Models: A Practical Approach to Development, Validation, and Updating.

[21] Vickers AJ, Elkin EB (2006). Decision Curve Analysis: A Novel Method for Evaluating Prediction Models. Med Decis Making.

[22] Collins GS, Moons KGM, Dhiman P (2024). TRIPOD+AI statement: updated guidance for reporting clinical prediction models that use regression or machine learning methods. BMJ.

[23] Cohen JF, Korevaar DA, Altman DG (2016). STARD 2015 guidelines for reporting diagnostic accuracy studies: explanation and elaboration. BMJ Open.

[24] Bujang MA, Adnan TH (2016). Requirements for Minimum Sample Size for Sensitivity and Specificity Analysis. J Clin Diagn Res.

[25] Almubayyidh M, Parry-Jones AR, Jenkins DA (2024). Development and internal validation of prehospital prediction models for identifying intracerebral haemorrhage in suspected stroke patients. BMJ Neurol Open.

